# How medical students experience empathy training? An Interpretative Phenomenological Analysis. Results from the “Empathize with me, doctor!” project

**DOI:** 10.15694/mep.2017.000111

**Published:** 2017-06-22

**Authors:** Vasileios Kiosses, Konstantina Symeonidou, Carolina Alexiou, Athina Tatsioni, Thomas Hyphantis, Ioannis Dimoliatis

**Affiliations:** 1 University of Ioannina; 2 Counselling Unit

**Keywords:** empathy, training, person-centered, interpretative phenomenological analysis

## Abstract

This article was migrated. The article was marked as recommended.

The aim of this study was to investigate how a detailed experiential empathy training program was experienced by medical undergraduates.

Twelve medical undergraduates completed a 60-hour, experiential, person-centered training, aiming at improving their empathic performance. After the completion of the training they were interviewed, using semi-structured interviews with open questions, while Interpretative Phenomenological Analysis (IPA) was used to extract the emerged themes.

IPA revealed one superordinate theme, i.e. the training as opportunity for actualization, and two subordinate themes, i.e. change in relating with others and self-concept reconstruction through introspection. The first subordinate theme had two components: change in relating with others regarding professional relationships and change related to personal relationships.

IPA of students’ transcripts revealed that the training was experienced by the students, as an opportunity to introspect, to deal with the obstacles occurring in their encounters with their patients, as a chance to eavesdrop to their inner needs and as a chance to actualize their potential.

## Introduction

Empathy is a widely known concept in the context of psychotherapy and counseling. It was first mentioned by Germans art aesthetics and it was later used in psychotherapy ( Iossifides & Iossifides, 2002). Buber (1937) coined the term empathy and Carl Rogers, the founder of Person Centered Approach (PCA), used it in counseling, stating that “empathy is the ability to sense the client’s world as if it were your own, but without ever losing the “as if” quality. This is empathy and this seems essential to therapy” ( Rogers, 1959, p. 98).

Empathy during doctor-patient relationships is not different than the empathy as described by Rogers. Asclepiades of Bithyna ( Cornelius Celsus, 1971), the “father” of what we call today molecular medicine, was the first who underlined the importance of empathy in these encounters. He used to say to his students to cure their patients safely, quickly and pleasantly (“ *ut tuto ut celeriter ut iucunde curet*”).

According to various researches empathy during doctor-patient relationship is linked with patients’ stress reduction ( Halpern, 2003) and facilitates a trustful and safe climate during clinical encounters ( Suchman, Markakis, Beckman & Frankel, 1997). Furthermore, an empathic healthcare professional creates a condition where patients feel more comfortable to speak about their concerns, about the disease they suffer from ( Fink, Sorensen, Engberg, Holm & Munk-Jorgensen, 1999). Moreover an empathic physician can identify more accurately somatoform symptoms occurring in patient ( Bellet & Maloney, 1991) and can possibly avoid burnout syndrome ( Anfossi & Numico, 2004).

A person can be trained in abilities such as empathy and the literature reveals the importance of such trainings in health care professionals. Specifically, different training programs have been developed in order to improve communication skills (including empathy) in these professionals. Different teaching methods, with a wide range in duration, have been already used in health care populations. These training programs improved significantly the empathic performance. (Blaire- Irvine et al., 2002; Rask, Jensen, Andersen, & Zachariae, 2009; Bonvicini et al., 2009; Shapiro, Lancee, & Richards-Bentley, 2009; Tulsky et al., 2011; Evans, Stanley & Burrows, 1993; Delvaux et al., 2005; Ozcan, Oflaz & Bakir, 2012; Razavi et al., 2002).

Previous studies have shown that empathy trainings can improve doctors’ expressed empathy ( Kiosses, Karathanos & Tatsioni, 2016). More specifically Riess, Kelley, Bailey, Dunn and Phillips (2012) showed that a brief intervention improved the empathic understanding of physicians as rated by patients. Another intensive training program for nursing staff increased the empathic statements and improved interactions with patients ( Herbek & Yammarino, 1990). In most of the trainings mentioned, empathy constitutes a part of a general communication skills training program. A systematic review of the literature of studies on doctor- patient communication skills training for medical students and physicians in mainland China revealed that such interventions can improve expressed empathy in the context of a communication skills training program ( Liu et al., 2014).

Empathy has been shown to improve therapeutic effect and the patients’ quality of life ( Halpern, 2003; Roter et al., 1997; Neumann et al., 2007; Spiro, 1992; Levinson, Roter, Mullooly, Dull & Frankel, 1997), as well as is linked with an increased sense of well-being ( Eikeland, Ornes, Finset & Pedersen, 2014).

Empathy becomes increasingly crucial in medical practice and during doctor patient encounters. In the “Learning Outcomes/ Competences for Undergraduate Medical Education in Europe: The Tuning Project (Medicine)” developed and approved by the MEDINE Thematic Network of about 100 European medical schools, validated by an external expert panel, and presented to the European Commission (2008), empathy is highlighted as a main professional attribute, incorporated in the Outcomes for Medical Professionalism ( Cumming & Ross, 2007; 2008). But, how can empathy be taught?

In an attempt to answer this question an experiential empathy training for medical undergraduates was developed, the “Empathize with me, Doctor!” ( Kiosses, Tatsioni, Dimoliatis, & Hyphantis, 2017). The experiential teaching method was far different from the usual teaching method used in medical schools. Greek medical schools tend to use specific teaching methods, like lectures, with the use of presentations in most cases. Experiential learning is learning by doing, including subjective experience in the learning process. The experiential way includes active participation of the students but the most significant element is the process of the personal development through learning. Yet another important component of the experiential learning is the relationship between the trainer and the trainee.

The Experiential Learning Model (ELM) was introduced by David Kolb (1984). According to this model, experiential learning relates to the meaning-making process of the trainee’s experience. In order to gain knowledge through experiential learning, the following conditions are required:

• The learner must be willing to be actively involved in the experience;
• The learner must be able to reflect on the experience;
• The learner must possess and use analytical skills to conceptualize the experience;
• The learner must possess decision-making and problem solving skills in order to use the new ideas gained from the experience.

This alternative way of teaching was used to train medical undergraduates in empathy, and this study explores how this teaching was experienced by them and how they felt during the training and how it was conceptualized.

Main aim of the study was to explore how medical undergraduates experienced their participation in the empathy training, through semi-structured interviews.

## Method

### Empathy Training

The “Empathize with me, Doctor” project lasted 60 hours distributed in three 20-hour workshops, three or four weeks apart from each other. The teaching method was experiential and the training included theory, personal growth and skills development. A detailed description of the “Empathize with me, Doctor” project is presented elsewhere ( Kiosses, Tatsioni, Dimoliatis, & Hyphantis, 2017).

### Interpretative Phenomenological Analysis

*Interpretative phenomenological analysis* (IPA), the experiential qualitative approach to research in humanistic and social sciences, was chosen for data analysis. The main reason for this was the fact that IPA focuses on the subjective experience of the trainees, as described by them, while at the same time the researcher adds his point of view ( Smith, 1996). The main aim of the IPA is to explore the meanings and the symbolization process of each participant, while participants share the same experience. IPA tries to understand experiences, and all the meanings and values hold for the participants.

The phenomenological base of the PCA and the phenomenological view of IPA, renders IPA as the most appropriate model to use, while trying to investigate or to better understand how trainees experienced the training. Authors try to deeper understand what the interviewees said, to read between the lines, to identify further details about their lived experiences, rather than to generalize the meaning of their experience. Aim of this analysis, and of the qualitative analysis in general, is the deeper study of phenomena through the interviewees’ frame of reference ( Smith, Flowers & Larkin, 2009). The researcher in this analysis has an active role in this process and has to identify the deeper meaning each participant deals with while describing the lived experiences ( Smith & Osborn, 2003).

The same shared experience for the participants is a necessary condition when conducting an IPA. IPA is concerned with exploring how participants make sense of that common experience ( Smith, 2011). As medical undergraduates, our trainees complied with the purposive sampling strategy of IPA ( Smith, Flowers & Larkin, 2009). The topic investigated during this analysis was the impact of the training on the students and how they experienced the training. The researcher seeks to describe rather than explain this experience and to understand how the experience was symbolized, perceived and organized into some relation to the students themselves. IPA is a method for seeking participants’ perceptions and understandings of experiences that are complex, poorly understood or previously unexplored ( Smith, 1996; Smith & Osborn, 2003; McCormack & Joseph, 2014).

The process of IPA involved the following stages. The main and most core stage was the repetitive listening of the interviews. This was followed by verbatim transcription of the interviews and the preparation of the first transcript. Repetitive and successive readings lead to paraphrasing the experience of each participant. Authors tried to identify key words above all transcripts, and furthermore to identify categories or emerging themes from the interviews. This process was repeated a few times for each transcript by the interviewer (V.K.). Finally, the raw data were categorized into meaning clusters and commonalities between the transcripts. Then the data revealed the superordinate theme, the subordinate themes and their components.

### Participants

Students from the fourth, fifth and sixth year of studies, from the University of Ioannina, Greece, were invited to participate in the training.

The final sample comprised from medical undergraduates from the University of Ioannina Greece, who successfully completed this experiential training in empathy. Twelve medical undergraduates (9 female, 3 male; aged 22 to 25, mean 23.3, standard deviation 0.9; at the fourth, fifth and sixth year of studies, 2, 8 and 2 respectively), completed successfully the experiential training in empathy.

### Interviews

The tool used for the analysis was the open, semi-structured interview. One week after the completion of the training, students were interviewed with semi-structured interviews, using the open-ended questions of Table 1.

After full explanation of the study, all participants signed a declaration of consent before the training. The interviews were not mandatory but recommended. None of them had ever participated in empathy training before. All of them accepted interviewing, the duration of which ranged from 18 to 43 minutes (mean 27.8, SD 8). All interviews were held by one of their trainers (V.K.) and, after obtaining the interviewees’ permission, were recorded with the help of a voice recorder.

## Results

One superordinate theme, the experience of the training as an opportunity for actualization, emerged, overarching two subordinated themes. The first referred to the change in relating with others and the second referred to the self-concept reconstruction through introspection.

Every participant described the training as a context in which the optimal conditions to listen themselves and their inner needs flourished. In some cases the training was described as the willingness and the attempt of the students to find a way to relate in depth with their patients and not only.

In the PCA framework described in Methods, the training in empathy created the condition for the trainees to develop their potential, to grow and change. The training offered the core conditions, so the trainees were free to develop their potential.

### Subordinate Theme I: Change in relating with Others (patients or not)

Most of the students experienced a change in relating with others. And this subordinate theme has two components. The first one is related to their encounters with the patients and the second refers to their encounters with friends and family. New knowledge and skills development are issues categorized to the first component, which helped them to treat patients differently, than they used to do. Enthusiasm is the main emotion expressed by the trainees, when talking about the training they took part in.

Specifically, they were referring a lot to the new way they learned to relate with their patients and the way they can fit in this relationship all the emotional components, each patient carries while dealing with a disease or while visiting a hospital’s emergency department. A 5 ^th^ year 23 year-old female trainee said:

*“I learned how to listen to patients. To understand their thoughts, their feelings. I never did this before. A patient may be afraid.. I didn’t understand this before. Now I can listen. I am more alert now. My “antennas” are activated.. I can observe the body language”*

This trainee referred to the broaden perception she had of the importance of the relationship between doctor and patient. She said that she could integrate all the emotional components of the patients, and not only the physical symptoms has a patient suffered from.

Another 5 ^th^ year female 23 year-old trainee said:

“ *I see myself different during my encounters with the patients. I changed my behavior.. the most simple thing I changed is the way I’m posing a question.. you know.* *I now avoid asking -why- questions.*”

This student discussed on the effect has the way doctor asks for symptoms. The trainee redefined the importance of not asking “why” questions, and how important is while asking for details to facilitate the patient. The trainee here discovered a whole new way to act, and this new way seems fascinating to her

Another 5 ^th^ year 24 year-old female trainee talked about new knowledge she gained and how she developed further skills which made her change her behavior, t:

*“I feel more comfortable now.. to communicate with a patient, I feel more qualified. I was always feeling insecure during these encounters. Now I can be more.. careful.. automatically. The way I introduce myself, the way I speak, the way I sit. This training was so helpful .. Before the training I knew that I had to be kind.. to get the information I want. In case I didn’t get it, I changed my behavior.. I was becoming strict. Never thought of the patient’s feelings. Never felt patient’s anxiety. Now.. when I come close to a patient.. I have in mind to look at his face and see if he’s scared, if he’s anxious. I want to listen to him, to understand how he feels. I want to know how he feels about all that’s happening to him; how he feels about the disease he suffers from”.*

And another female 6 ^th^ year 24 year-old trainee said:

“ *I think I now treat them differently. Now I know how important is to let the patient talk.. you know.. these two “golden minutes”. I saw it happening in front of my eyes.. how helpful it is for the patient. I know it is worth happening. In most times when I focus on the psyche of the patient, they are open up more. Sometimes they tell me things that are not recorded in the medical history*”.

These trainees learned how simple behavior changes can make them empathic Trainees stated how the training helped them change specific aspects of their behavior towards the patients and hence how facilitative this is.

This subordinate theme, as we have already mentioned, has another component. The change of the behavior related to their personal relationships, and not only to their encounters with patients. Eight of the trainees referred to the changes towards their friends. One female 4 ^th^ year 22 year-old said:

“ *I realized a few things. Through this process I learned that people have many aspects. I knew it cognitively but I saw it happening. I changed the way I see people. You don’t judge them from something.. sure there’s something beautiful in each. I learned how to give chances without judging.. easier said than done. I am really excited. In general I was curious about the way I relate to others. I’m so happy I discovered another way. I have a lot to do now..*”

Trainees discovered that this new way of being, this alternative way of communication affects every aspect of relationship. They realised that during their personal relationships they can act differently, more empathic, a way which is unique, relieving and facilitating. A female 6 ^th^ year 25-year old trainee said:

*“I like a lot this training. I think that it is helpful.. not only in how I can be empathic with my patients but in my personal encounters as well. I can hear clearly what they say to me, how they feel. I can understand their feelings. I’m not involved in hospital work currently, but I am working on it with my friends and family”.*

Another male 4 ^th^ year 22-year old trainee said:

“ *Empathy is not only connecting with patients, but it is about every relationship. Through this training, I had a second thought.. on how I see things. I did some connotations while we were trained. Empathy for me is a skill applied in every relationship. Doctor-patient relationship is only one aspect of relationship.*”

Trainees clearly stated the importance of the empathy in every aspect of their relationship.

### Subordinate Theme II: Self-Concept Reconstruction through Introspection

All trainees, without exception, discovered new aspects of themselves. All of them were referring to the training as an opportunity to identify who they were to assimilate with the others, to reconstruct the way they see themselves.

The training offered to the trainees the condition to introspect, to visualize themselves, to ask themselves. So they had the chance to identify and decode their behaviors. They learned how to be empathic through an empathic atmosphere. They experienced it. A 5 ^th^ year male 24-year old said:

“ *It was a surprisingly rich experience for me. I sorted my thoughts.. I already knew a few things about me, but I couldn’t fit them in my mind. See.. I can keep from this process the time we had for ourselves.. the silence.. the threads.. Before the training I was losing this time. It helped me to respect myself, and thus respect the others. For me it wasn’t a priority to respect myself.. now it is.. the training helped me a lot*”

For this student, his self-concept did not include self-respect. Previous the participation in the training he had learnt that he had no such right. Through the training, he discovered that self-respect is a way to take care of himself.

Another 5 ^th^ year female 23-year old student said:

“ *It was a personal journey for me.. I am not used to talk to people about my feelings.. the fact that I was moved in front of so many people it was strange or even threatening at the beginning .. liberating afterwards .. finally it was cool. It was about how I can be empathic towards myself. It’s not that I found out things for me I didn’t know. I had the information.. but.. I found myself able to share this information with the others. This is a huge step for me.. it’s hard for me to share such things. I am emotional but I would never show it. Now I learned that it is OK to show it..*”

This emotional aspect of this student was repressed. She could not accept that aspect of herself. We do not have to interpret why this happened, but we can see that the training was not threatening for her, offered her the condition where all feelings are welcomed. As the others welcomed her feelings, she was able, not only to accept them, but to share and feel safe with that.

A 6 ^th^ year female 24-years old said:

“ *I learned a lot about me, but due to the intensity of the training I couldn’t think of them as much as I wanted. Well, the most important thing.. I incriminated some aspects of me.. I had the chance to redefine them. Through the trainers.. through what I heard from the group.. I’m trying to be a better person every time.. maybe this is why I focus on my negative aspects. The training helped me not to incriminate my smile, my positive energy. I wish I were more open to people*”.

The training helped this student to integrate all these “negative aspect” she thought she had. It helped her to reconstruct her self-concept, including the new aspects she discovered through the training process.

A 5 ^th^ year 24-years old female said:

“ *I think that the main aim of the training was to introspect, to see ourselves. It was nice. I can see a change in me. I thought that I will always be weak to recall facts. I realized that the power is within.. when you “delve” into these facts. I thought that I would never change, but finally I am changing. I think that this is the most important thing I’ve learned.. I’ve learned to appreciate the silence.. for me it was a process.. but mostly.. I became strong.. and realized that I can be stronger*”.

A whole new dimension was revealed to this student, when she was offered through the training group not only the opportunity but the appropriate safe environment, to introspect, and to recall facts she was afraid to recall. According to her self-structure, weakness was one of her aspects. When she felt that the group was not threatening for her, she introspected, and discovered the strength she never knew she possessed.

## Discussion

Twelve medical students completed an experiential training aiming at improving their expressed empathy. Using interpersonal phenomenological analysis (IPA), authors analyzed the data collected through semi-structured interviews which were conducted one week after the completion of the training. The analysis revealed one super ordinate theme, and two subordinate themes. The super ordinate theme was “the experience of the training as an opportunity for actualization” and the subordinate themes were “change in relating with others” and “reconstruction of the self-concept through introspection”. To the best of our knowledge this is the first study in the literature exploring how such training is experienced by medical undergraduates.

The participants described the training as an opportunity to actualize, to develop their potential, to investigate who they are or what they are able to do. This is how group counseling works, but with the difference that they were invited to develop empathic skills as well. Students described the training as a chance to introspect, as a chance to deal with the obstacles occurring in their encounters with their patients, as well as a chance to listen to their inner needs.

Although the training was about how to be empathic during the clinical encounters and the relationship with patients, trainees referred to the change they noticed during their encounters with friends and family. An environment full of empathy, unconditional positive regard and genuineness, facilitated them to talk about their concerns, to hear their inner voices, and to deal with aspects of themselves they were not aware of, or they had never dealt with before.

Undergraduates reported a greater acceptance and understanding of any thought regarding their professional experience. The fact that their experiences were matched created a sense of relief, which made them feel safe, and accompanied. This agrees with the theory of the PCA that the self-concept is linked to our experience and influences our perception of the world and the perception of oneself. According to Rogers (1951) “ *as the individual perceives and accepts into his self- concept more of his organic experiences, he/she finds that he is replacing his/her present value system -based extensively on introjections which have been distortedly symbolized- with a continuing organismic valuing process*” (p. 522).

Trainees felt that being empathic, is not only a development of communication skill, but it goes beyond that. It is not a technique; it is about stepping into other people’s world. They realized how important is to introspect and satisfy their inner needs. All these processes are included in the process of being empathic and that differentiates empathy from trying to develop communication skills. They realized that when relate with someone, whether he/she is a patient, friend, spouse, parent, colleague, you reflect yourself, and the other reflects on you. So, through this intervention, trainees tried to explore how they relate to others and to redefine the way they introspect and share experiences. It is also interesting and noteworthy that trainees described these specific attitudes they redefined after the training, about their behavior during their relationship with the patients. They described for example the importance of avoiding asking “why” questions and the importance of the “golden minutes”. “Why” questions tend to be aggressive or even hostile and put people on the defensive. For example “Why are you still smoking, despite you know you’re suffering from breathing issues?” is not a sensitive approach. A friendlier way to say the same thing would be “I’m concerned about your health issues and I want us to find a way to help you quit smoking” ( Leigh, 2014). Additionally the “golden minutes” are about two minutes for the patients, at the beginning of the clinical encounter, where patients can talk about the symptoms or whatever else they think is important. These minutes at the start of the encounter are essential in making the patient feel safe, secure and create a facilitating atmosphere. In most cases doctors ask too much when they hear the first symptom. So there is not enough time for the emotional components of the symptoms ( Benbassat & Baumal, 2004).

Qualitative research in medical undergraduates reveals that there are several obstacles in performing in an empathic way. Undergraduates suggested that being and becoming a professional, using cynicism as a coping strategy or the difficulty to balance between distance and empathy influence their empathy during their encounters with patients ( Eikeland, Ornes, Finset & Pedersen, 2014).

Another qualitative research in fifth year medical undergraduates revealed their demand for opportunities to discuss about the doctor- patient relationship and their willingness to develop empathic relationships. The same research revealed that undergraduates are experiencing anxiety about handling patients’ psychological characteristics ( Noqueira- Martins & Noqueira- Martins, & Turato, 2006).

Previous study demonstrated that physicians participating in communication skills training based in mindfulness and focused on self -awareness, improved personal well- being, including burnout ( Krasner et al., 2009). Participants in Krasner’s et al (2009) study experienced positive changes in empathy and psychosocial beliefs.

A limitation of our study might be that findings of our research are unique to the current sample, just as every qualitative method. As IPA stands, every attempt for understanding subjects’ world, will always be subject to a degree of mediation according to the researchers’ own epistemological stance, or furthermore frame of reference. Another limitation of the study is the fact that the interviewer was the facilitator and trainer as well. This could possibly would led to subjective outcomes, as perceived by the interviewer during the training.

“What is most personal is most universal”. This is a famous quote by Carl Rogers, the founder of PCA ( Rogers, 1961). What does he mean by that? When people share experiences, in a very or not so strange way, their words seem familiar with us. Personal experience is universal in a way nothing else can be. What is real, our direct experience turns out to be universal. This is how at the heart of suffering we find compassion or empathy for others. In such words the most hidden is the most common. Feelings that we have, we tend to experience them as unique. In fact it is unique the way we experience them, but these feelings are actually quite common. This is something we clearly see in our interpretative phenomenological analysis. Twelve medical undergraduates were trained in empathy in an experiential way. They learned, through experience, how to be empathic with their others, patients or not. The training included theory, skills development and personal growth. They learned how to be empathic, through an empathic process. Theory and practice were combined and we’ve tried to investigate how this training was experienced by them.

Future research should seek to investigate the duration of the feelings expressed by the trainees. All of them were impressed by this experiential training, and it would be interesting to search whether this enthusiasm lasts more than right after the completion of the training, or whether the changes they were referring to, are long lasting. Similarly, it would be of an exceptional interest to investigate how empathy trainers experience the group process and each participant’s introspection.

## Take Home Messages

Through the experiential training aiming at improving their empathic performance, participants expressed that the training facilitated them not only to gain knowlegde on how to be empathic, but furthermore to increase their self-awareness and self-competence. This experience of their participation was a chance to introspect and hence to actualize their potential.

## Notes On Contributors

Vasileios Kiosses is licensed psychologist and PhD candidate in Medical Education, Medical School, University of Ioannina, Greece.

Konstantina Symeonidou is mental health counselor trained in Person-centered counseling working in private practice.

Carolina Alexiou is mental health counselor trained in Person-centered counseling working in private practice.

Athina Tatsioni is Assistant Professor, Department of Internal Medicine, Medical School, University of Ioannina, Greece.

Thomas Hyphantis is Professor, Department of Psychiatry, Medical School, University of Ioannina, Greece.

Ioannis Dimoliatis is Associate Professor, Medical Education Unit, Department of Epidemiology, University of Ioannina, Greece.

## Appendices

**Table 1.** Questions used in the interviews

• Have you ever thought that the relationship with your patients is something you need to develop, before you knew about the empathy training? • How you experienced the training? • Did you trace any changes in your encounters with your patients? • Which was the most important thing you learnt? • Would you like to be taught in the same experiential way, other medical courses? • Is there anything you would change in the training? • During the training, did you search further information about empathy? Did you spend time for searching?
