# Supplementary material for: How medical students experience empathy training? An Interpretative Phenomenological Analysis. Results from the “Empathize with me, doctor!” project
Source: MedEdPublish (2016). 2017 Jun 22;6:111. [Version 1] doi: 10.15694/mep.2017.000111 (PMC10885267; doi:10.15694/mep.2017.000111)
Supplement: Peer review history

# Reviewer report: Reviewer response for version 1

**DOI:** 10.21956/mep.19272.r28013

**Published:** 2018-11-24

**Authors:** Peter Dieter

**Affiliations:** 1 AMSE

This review has been migrated. The reviewer awarded 3 stars out of 5 This study investigated how an empathy training program was experienced by medical undergraduate students. The result of the study was that the students gained knowledge on how to be empathic and that the training program increased their self-awareness and self-competence.The study has many limitations: only 12 students from one university took part in the study; the study was not repeated in subsequent years to see if there was a long-lasting effect; the study did not distinguish between students from 4th, 5th and 6th year.

Reviewer Expertise:

NA

No decision status is available

# Reviewer report: Reviewer response for version 1

**DOI:** 10.21956/mep.19272.r28016

**Published:** 2017-07-12

**Authors:** Theofanis Sitzanis

**Affiliations:** 1 University of Ioannina

This review has been migrated. The reviewer awarded 5 stars out of 5 Reading this almost four years after the training, I can remember exactly how it felt, the moments of realisation and clarity, the "breakthroughs" that helped me tidy up my conception of the world in my mind, and thus, my conception of relationships between people and between doctors and patients. The nature of the training, which relied heavily on personal experience rather than memorising theories and lectures, makes it possible to "re-live" the important aspects of it whenever I need to, and one can't help but wonder how effective it would be if more "classic" and established ways of teaching could benefit from such an approach.In conclusion, this specific seminar (using the term "seminar" loosely) was one of the fondest memories I have from medical school, leaving me with nothing but invaluable tools in my everyday medical practice and life.

Reviewer Expertise:

NA

No decision status is available

# Reviewer report: Reviewer response for version 1

**DOI:** 10.21956/mep.19272.r28015

**Published:** 2017-06-25

**Authors:** Tania Chaldaiopoulou

**Affiliations:** 1 University of Ioannina

This review has been migrated. The reviewer awarded 5 stars out of 5 As a fifth grade medical student at University of Ioannina, I had the chance to attend to the project "Empathize with me doctor". At first, I was really hesitant about the meaning and the effectiveness of empathy in patient's treatment. I thought that only clinical skills and drugs would be adequate for a doctor in order to provide the best medical care. After the end of the project, I had realized that empathy is one of the greatest skills for a doctor. It contributes to a better doctor - patient communication and helps the doctor to understand more about the patient's condition. Also, thrοugh examples and continuing practice, we have learned how to interprete the body language and make the patient feel comfortable with us. We tried to understand their feelings and not judge them, we put ourselves in their shoes.I believe that "Empathize with me doctor" is a great project that prepares medical students for their future career and I strongly recommend it.

Reviewer Expertise:

NA

No decision status is available

# Reviewer report: Reviewer response for version 1

**DOI:** 10.21956/mep.19272.r28012

**Published:** 2017-06-23

**Authors:** Subha Ramani

**Affiliations:** 1 Harvard Medical School, Brigham and Women's Hospital

This review has been migrated. The reviewer awarded 4 stars out of 5 Empathy in patient care is very important and it is always disappointing to review studies that show declining empathy with increasing level of training. I enjoyed reading the overview of empathy, its definitions, origins and history. I completely agree with the authors that this is a quality most important to the doctor-patient relationship. Rapport with patients enhances patients’ trust in their doctors, which in turn ensures higher levels of adherence to medical recommendations. There is room for optimism that empathy can be taught and I concur that this is a skill more effectively ingrained through experiential learning strategies. Therefore Kolb’s model is an excellent framework to apply to their training. IPA is a very appropriate research approach to explore the perceptions of trainees regarding the training and derive meaning. Since self-actualization is considered an important aspect of internal motivation, it is interesting that this theme was generated from these data. The write up of the introduction and results sections is very clear. The themes they obtained are illuminatingMy constructive comments include the following:- I wish there had been a more detailed description of the curriculum at least in an appendix section- More details of the methods and measures to ensure rigor would be helpful to those wishing to replicate the study- The discussion is a section that offers some literary license to authors as long as the data back up their interpretations. I wish I saw more of the link between their results and abstract constructs such as self-determination theory or self-efficacy or even professional identity. While the authors have summarized their results well and referenced previous reports, I would liked to have seen them reach beyond the results into deeper constructs. Maybe next study.Thank you for the opportunity to review this paper.

Reviewer Expertise:

NA

No decision status is available

# Reviewer report: Reviewer response for version 1

**DOI:** 10.21956/mep.19272.r28014

**Published:** 2017-06-22

**Authors:** Trevor Gibbs

**Affiliations:** 1 AMEE

This review has been migrated. The reviewer awarded 3 stars out of 5 A very interesting paper that attempts to tackle a rather difficult question related to the value of a course in empathy, I felt that it brought out some very interesting points for discussion and showed the immediate positive effects upon the students.Personally I would have appreciated seeing the content and methodology of the course, although I realise it is referenced accordingly. I would have liked to here whether there appeared to be any difference between the younger students and those in later years, given the basis of this paper relates to experiential learning and of course, it would be better to have more students and follow them through subsequent years, demonstrating the longer term value.However despite these reservations, I do feel that it is a paper worth reading to those involved in curriculum development

Reviewer Expertise:

NA

No decision status is available
